# Infusion of young donor plasma components in older patients modifies the immune and inflammatory response to surgical tissue injury: a randomized clinical trial

**DOI:** 10.1186/s12967-025-06215-w

**Published:** 2025-02-14

**Authors:** Brice Gaudilliere, Lei Xue, Amy S. Tsai, Xiaoxiao Gao, Tiffany N. McAllister, Martha Tingle, Gladys Porras, Igor Feinstein, Dorien Feyaerts, Franck Verdonk, Maximilian Sabayev, Julien Hedou, Edward A. Ganio, Eloïse Berson, Martin Becker, Camilo Espinosa, Yeasul Kim, Benoit Lehallier, Esther Rawner, Chunmiao Feng, Derek F. Amanatullah, James I. Huddleston, Stuart B. Goodman, Nima Aghaeepour, Martin S. Angst

**Affiliations:** 1 Department of Anesthesiology, Perioperative and Pain Medicine, Stanford University School of Medicine, 300 Pasteur Drive, Stanford, CA 94305 USA; 2 Department of Pediatrics, Stanford University School of Medicine, Stanford, CA USA; 3 Department of Biomedical Data Science, Stanford University School of Medicine, Stanford, CA USA; 4 Department of Anesthesiology and Intensive Care, Hôpital Saint-Antoine, Assistance Publique-Hôpitaux de Paris, Paris, France; 5 Institute for Computational and Mathematical Engineering, Stanford University, Stanford, CA USA; 6 Institute for Visual and Analytic Computing, University of Rostock, Rostock, Germany; 7 Alkahest Inc, San Carlos, CA USA; 8 Department of Orthopedic Surgery, Stanford University School of Medicine, Stanford, CA USA

**Keywords:** Aging, Elderly population, Inflammaging, Inflammation, Immune cells, Tissue injury, Proteomics, Surgery, Young plasma, Tissue injury

## Abstract

**Background:**

Preclinical evidence suggests that young plasma has beneficial effects on multiple organ systems in aged mice. Whether young plasma exerts beneficial effects in an aging human population remains highly controversial. Despite lacking data, young donor plasma infusions have been promoted for age-related conditions. Given the preclinical evidence that young plasma exerts beneficial effects by attenuating inflammation, this study examined whether administering a young plasma protein fraction to an elderly population would exert anti-inflammatory and immune modulating effects in humans, using surgery as a tissue injury model.

**Methods:**

This double-blind, placebo-controlled study enrolled and randomized 38 patients undergoing major joint replacement surgery. Patients received four separate infusions of a plasma protein fraction derived from young donors, or placebo one day before surgery, before and after surgery on the day of surgery, and one day after surgery. Blood specimens for proteomic and immunological analyses were collected before each infusion. Based on the high-content assessment of circulating plasma proteins with single-cell analyses of peripheral immune cells, proteomic signatures and cell-type-specific signaling responses that separated the treatment groups were derived with regression models.

**Results:**

Elastic net regression models revealed that administration a young plasma protein fraction significantly altered the proteomic (AUC = 0.796, *p* = 0.002) and the cellular immune response (AUC 0.904, *p* < 0.001) to surgical trauma resulting in signaling pathway- and cell type-specific anti-inflammatory immune modulation. Affected proteomic pathways regulating inflammation included JAK-STAT, NF-kappa B, and MAPK (*p* < 0.001). These findings were confirmed at the cellular level as the MAPK and JAK/STAT signaling responses were diminished and IkB, the negative regulator of NFkB, was elevated in adaptive immune cells.

**Conclusion:**

Reported findings provide a first proof of principle in humans that a young plasma protein fraction actively regulates inflammatory and immune responses in an elderly population. They provide a solid rationale for elucidating active principles in young plasma that may be of therapeutic benefits for a range of age-related pathologies.

**Trial registration:**

ClinicalTrials.gov, NCT 03981419.

**Supplementary Information:**

The online version contains supplementary material available at 10.1186/s12967-025-06215-w.

## Background

Substantial evidence suggests that young plasma exerts beneficial effects in multiple tissues and organ systems of aged mice. However, it is highly controversial whether administrating young plasma exerts beneficial effects in an aging human population. Despite the absence of data, young donor plasma infusions have been promoted as an unproven treatment for various age-related conditions, which led the FDA to issue a warning regarding such use [1–3].

Interest regarding the potential benefits of young plasma for reversing age-related biology and disease burden was triggered by rodent experiments using a heterochronic parabiosis model. In this model, a young and an old animal are surgically connected to share their circulation. In 2005, Conboy et al. demonstrated that progenitor cell activity in muscle and liver of older mice could be restored by circulating factors that change with age [4]. The beneficial effects of heterochronic parabiosis on the brain of aged animals were subsequently demonstrated, as circulating factors increased neurogenesis, enhanced neuronal plasticity, and improved cognitive function [5, 6]. Notably, in an Alzheimer’s disease model, young plasma targeted molecular pathways involved in learning, memory, and inflammation while improving cognitive function [7]. A series of heterochronic parabiosis studies demonstrated additional beneficial effects in other tissues and organs of aged mice, including enhanced vascular remodeling in the brain, improved bone healing in early fracture, and restoring kidney tissue architecture [8–10].

The strongest evidence that young human plasma may exert beneficial effects comes from experiments conducted on aged mice injected with umbilical cord plasma, which increased synaptic plasticity and improved cognitive function [11]. These effects were partially mediated by tissue inhibitor of metalloproteinase 2 (TIMP2). TIMP2 exhibits anti-inflammatory properties in microglia cell lines stimulated with lipopolysaccharide [12]. Studies conducted using heterochronic parabiosis also indicate that the decreased expression of proinflammatory cytokines and the regulation of the sentinel NFkB pathway are associated with beneficial effects of young plasma [7, 8]. These findings are consistent with the significant pathophysiological impact of inflammaging, a low-grade proinflammatory phenotype associated with age, on age-related disease burden, including atherosclerosis, obesity, and type-2 diabetes [13–15].

Given the significance of inflammaging and the preclinical evidence indicating that young plasma exerts beneficial effects by attenuating inflammation, the present study was conducted in an elderly population undergoing major surgery to examine whether the administration of a young plasma protein fraction mitigates inflammatory and immune responses in a human tissue injury model. Surgery elicits an archetypical inflammatory and immune response, and variations of this response have been associated with delayed recovery [16, 17]. Studies in trauma patients echo these findings, indicating that an exaggerated and prolonged inflammatory response increases morbidity and mortality [18, 19].

## Methods

This study was approved by the Institutional Review Board of Stanford University School of Medicine and registered at ClinicalTrials.gov (NCT 03981419). Written informed consent was obtained from all study participants before study enrollment. We followed the Consolidated Standards of Reporting Clinical Trials guidelines.

The primary objective of this randomized and double-blind phase 2a trial was to evaluate the hypothesis that administering a plasma protein fraction derived from young donors would alter the immune response to surgical injury and exert anti-inflammatory effects in an elderly population.

### Participants and study design

**Fig. 1:**
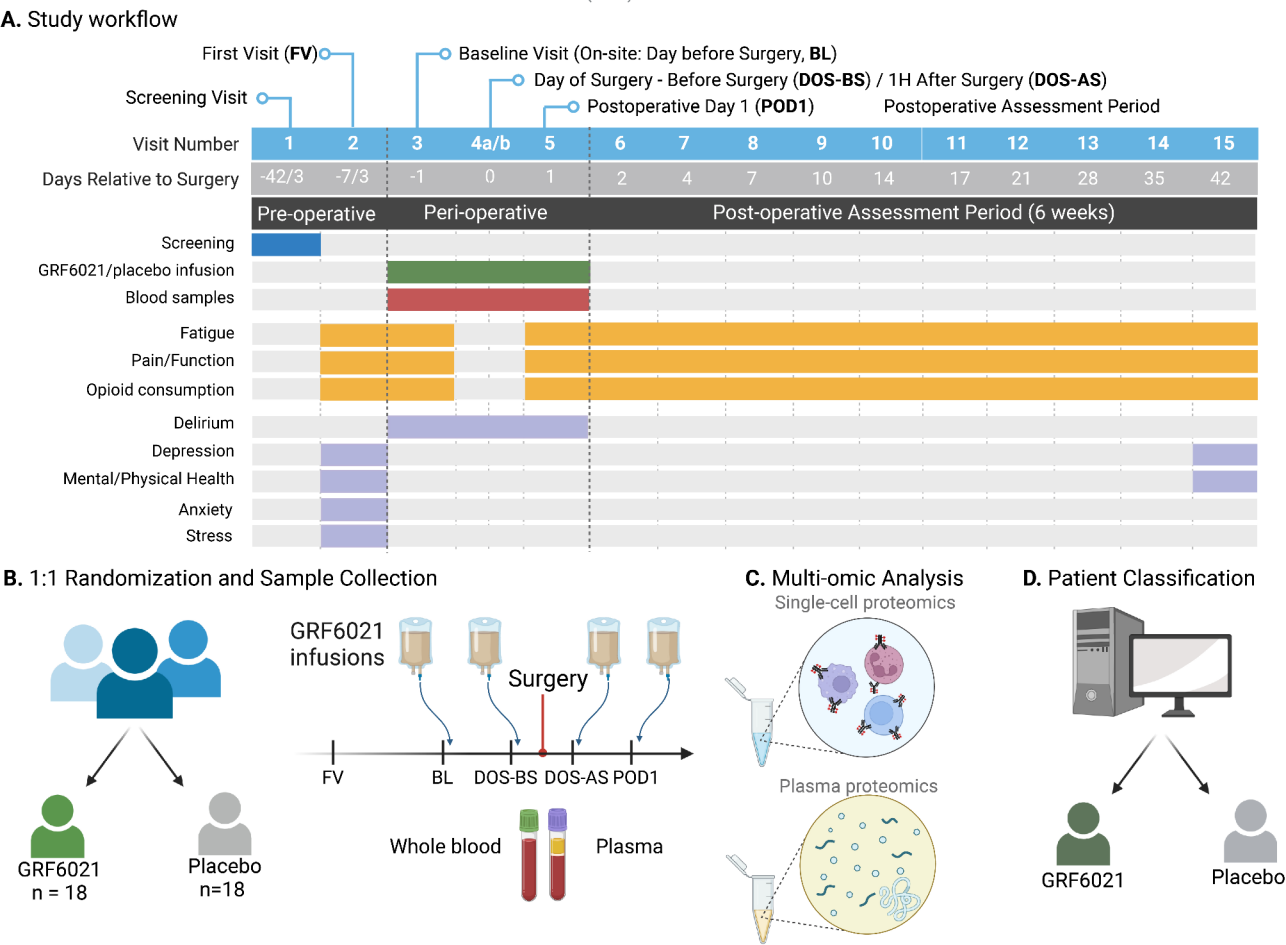
Study overview. (**A**) Thirty-six patients undergoing total hip or knee arthroplasty completed the study and were included in the final analysis. The three major clinical outcomes, fatigue and associated impairment of daily functioning, pain and function of the affected joint, and opioid consumption were serially assessed starting one week before and ending six weeks after surgery. Delirium was screened for during the perioperative period. Additional clinical assessments included physical and mental health, depression, anxiety, and stress. (**B**) Eighteen patients either received four sequential infusions of the young plasma protein fraction GRF6021, or saline placebo one day before surgery (BL), on the day of surgery before (DOS-BS) and after surgery (DOS-AS), and on post-operative day 1 (POD1). Blood specimens were collected serially before starting each infusion. (**C**) Isolates from whole blood samples were used to quantify and characterize single-cell and plasma proteomics. (**D**) Patient classification was performed using an Elastic Net machine learning algorithm to identify single-cell and plasma proteomic features separating the two treatment groups.

Patients scheduled for primary total knee or hip arthroplasty at Stanford Medicine were screened for eligibility. Patients were randomized to receive four separate infusions of GRF6021, an FDA approved 5% plasma protein fraction derived from young donors, or saline placebo, one day before surgery, before and after surgery on the day of surgery, and one day after surgery. Blood specimens for proteomic and immunological analyses were collected before starting each infusion (Fig. 1). Secondary clinical outcomes were assessed before and over the course of six weeks after surgery. They included fatigue and associated impairment of daily functioning, pain and associated opioid consumption, and functional impairment of the affected leg. The list of inclusion and exclusion criteria is provided in supplemental materials.

### Study intervention

GRF6021, manufactured by Grifols Therapeutics, Inc. (Clayton, North Carolina, US), is an iso-oncotic and purified 5% plasma protein fraction containing albumin and depleted of coagulation factors and gamma globulins. Each 100 ml contains 5 g of selected plasma proteins buffered with sodium carbonate and stabilized with 0.004 M sodium caprylate and 0.004 M acetyltryptophan. The average age of subjects providing blood for the production of GRF6021 is 35 years.

### Randomization and blinding

Patients were randomized 1:1 in blocks of eight and stratified by sex using Research Randomizer [20]. Patients, and all study personnel interacting with the patients were blinded. GFR6021 or saline placebo was stored and delivered by the pharmacy, and administered by an unblinded study nurse not interacting with the patient. The infusion bags and all tubing were concealed by a non-transparent cover, rendering it impossible to discern the color of the infused solution.

### Surgery and anesthesia

Hip and knee arthroplasties were performed by one of three surgeons using standard surgical approaches. The anesthetic management followed routine clinical protocols and was driven by the care providing team and patient-specific requests. Patients either underwent neuraxial, general, or a combination of neuraxial and general anesthesia (Table 1). All patients undergoing knee arthroplasty were offered a continuous adductor canal block for intra and postoperative pain control. The only study-related restriction was the preclusion of using ketamine, corticosteroids, and lidocaine as a continued intravenous infusion in the perioperative period because of their potential to modulate the immune response.

**Table 1:** **Patient and Clinical Characteristics**

DEMOGRAPHICS AND PRESURGICAL CHARACHTERISTICS^1^		
	GRF6021	Placebo
Sex (male/female)	8/10	10/8
Age (years)	71.5 (64.3–74.8)	64.5 (57.3–71.3)
Body mass index (kg/m^2^)	28.9 (25.7–33.2)	28.7 (25.7–31.1)
Race (number)		
African American	1	0
Asian Caucasian	1 9	0 15
Pacific Islander Unknown or not declared	1 6	1 2
Surgical Recovery Scale (17–100)	79.5 (65.4–84.2)	89.0 (78.8–95.9)
Western Ontario and McMaster Universities Osteoarthritis Index		
Pain (0–40)	22.0 (8.0–24.0)	15.0 (12.0-19.5)
Functional impairment (0–60)	30.0 (22.0–42.0)	22.5 (14.3–34.8)
36-Item Short Form Health Survey (z-score)		
Physical Composite Score Mental Composite Score	33.6 (26.7–36.4) 60.3 (49.0–63.0)	31.5 (29.0-39.5) 59.2 (54.4–62.8)
Beck Depression Inventory (0–63)	4.0 (2.0-8.3)	4.0 (1.0–6.0)
Profile of Moods Anxiety Scale (0–36)	4.5 (1.0-12.3)	5.0 (2.0–8.0)
10-Item Stress Scale (0–40)	9.0 (6.3–14.0)	10.0 (5.3–16.8)
**ANESTHESIA AND SURGERY**		
Arthroplasty (knee/hip)	12/6	12/6
ASA class (1–5)	3 (2–3)	2 (2–3)
Anesthetic technique^2^ (number)		
General & Neuraxial	1	1
General	3	5
Neuraxial	14	12
Times (min)		
Surgery	105 (102–119)	102 (96–114)
Anesthesia	180 (158–210)	165 (158–195)
Blood loss (ml)	100 (82–150)	100 (60–190)
Intraoperative fluids (ml)	1000 (850–1225)	1000 (825–1300)
Opioid use^3^ (mg)		
Day 1 after surgery Day 2 after surgery	2.3 (1.9–3.1) 1.9 (0.9–4.1)	3.4 (1.7–5.2) 2.3 (1.8–4.5)
Time to discharge (days)	2 (2–2)	2 (2–2)

1) Values represent median and interquartile range

2) A continuous adductor canal block was established preoperatively in 11/12 and 10/12 patients undergoing knee arthroplasty in the active treatment and placebo group, respectively

3) Postoperative intravenous hydromorphone equivalence

### Clinical outcomes

The study was designed to detect significant differences in molecular outcomes. The rationale for assessing clinical outcomes was to observe directional changes based on group assignment. Fatigue and associated impairment of daily functioning was assessed with the Surgical Recovery Scale (SRS) [21]. Pain and function of the operated leg were assessed with an adapted and validated version of the WOMAC scale [17]. Opioid consumption was quantified as the daily intake of intravenous hydromorphone equivalents (mg), using a conversion table to allow for group comparison [22]. The assessment of all clinical outcomes is described in detail in supplemental materials.

### Proteomic assay

Blood was collected by venipuncture into EDTA tubes, which were inverted 5 times before being placed on ice. Centrifugation started within 30 min of blood collection using a double-spin procedure. Tubes were spun for 10 min at 2000 x g at room temperature (16–24 °C), plasma supernatant was then transferred to polypropylene tubes, which were spun for 10 min at 2500 x g at room temperature. After centrifugation, plasma supernatant was transferred in aliquots to cryovials and stored at -80 °C.

Plasma samples were assayed by SomaLogic (Boulder, CO) using a highly multiplexed, aptamer-based platform measuring 6,596 unique protein targets [23, 24]. The assay quantifies proteins over a wide dynamic range (> 8 log) using chemically modified aptamers with slow off-rate kinetics (SOMAmer reagents). Each SOMAmer reagent is a unique, high-affinity, single-strand DNA endowed with functional groups mimicking amino acid side chains. Protein levels were expressed as relative fluorescence units. The proteomic assay used pooled calibrator replicates to estimate the coefficient of variation as a reproducibility metrics. The median coefficient of variation was 3.8% (1.9–8.8%; 10th and 90th percentile) across all plates, indicating excellent assay performance. A more detailed description of the assay is provided in supplementary materials.

### Mass cytometry

Whole blood was collected in sodium-heparin tubes and processed within 30 min. Samples were fixed in Smart Tube buffer (Smart Tube Inc., San Carlos, CA) and stored at -80 °C [25]. To process samples with mass cytometry, each sample was thawed, and erythrocytes lysed using Thaw-Lyse Buffer (Smart Tube Inc., San Carlos, CA), before barcoding with palladium isotopes, and staining with 26 metal-conjugated surface antibodies targeting phenotypic markers and 11 intracellular antibodies targeting functional markers using established protocols (Supplementary Table 1) [26]. Antibodies were chosen based on previous work highlighting the importance of targeted cell-specific functional differences for clinical recovery outcomes [17, 25, 26]. Barcoding details are provided in supplemental materials.

Gating was performed using Immune Atlas [27]. The gating strategy is depicted in Supplementary Fig. 1. Thirty-one cell-types were considered for subsequent analyses: granulocytes, eosinophils, basophils, B cells, CD56brightCD16- natural killer (NK) cells, CD56dimCD16 + NK cells, CD4 + CD45RA- CD62L + central memory T cells (CD4Tcm), CD4 + CD45RA + CD62L + T cells (CD4Tnaive), CD4 + CD45RA + CD62L + resident memory T cells (CD4Trm), CD4 + CD45RA- CD62L- effector memory T cells (CD4Tem), CD4 + Tbet + T cells (Th1), CD4 + Tbet + CD45RA- T cells (Th1mem), CD4 + Tbet + CD45RA + T cells (Th1naive), Th2, Th17, CD4 + CD25 + FoxP3 + T cells (Treg), CD4 + CD25 + FoxP3 + CD45RA- T cells (Tregmem), CD4 + CD25 + FoxP3 + CD45RA + T cells (Tregnaive), CD8 + CD45RA- CD62L + central memory T cells (CD8Tcm), CD8 + CD45RA + CD62L + T cells (CD8Tnaive), CD8 + CD45RA + CD62L + resident memory T cells (CD8Trm), CD8 + CD45RA- CD62L- effector memory T cells (CD8Tem), TCRγδ T cells, NK T cells, CD14 + CD16- classical monocytes (cMCs), CD14-CD16 + non-classical monocytes (ncMCs), CD14 + CD16 + intermediate monocytes (intMCs), monocytic myeloid-derived suppressor cells (M-MDSCs), myeloid dendritic cells (mDCs), and plasmacytoid dendritic cells (pDCs).

Cell frequencies were extracted as a percentage of gated live mononuclear cells (cPARP − CD45 + CD66−) for mononuclear cells. Neutrophil frequencies were expressed as a percentage of gated live cells (cPARP). Frequency features were expressed as differences in cell frequency relative to the baseline, the time point of blood collection before the first administration of the young plasma protein fraction or saline placebo one day before surgery.

The intracellular expression of phospho-(p)STAT1, pSTAT3, pSTAT5, pSTAT6, pNF-κB, pMAPKAPK2, pP38, prpS6, pERK1/2, pCREB, and total IκBα was quantified per single cell. Baseline signaling activities was expressed as the median signal intensity (arcsinh transformed value). Signaling changes were expressed as the difference in median signal intensity (arcsinh ratio) when compared to BL. Due to the considerable variance in basal signaling states among various cell types, it is common and advantageous to express cell-specific intracellular signaling characteristics in comparison to baseline. This normalization allows for comparing signaling features across different cell types. By extension, all features incorporated in the EN analysis of the mass cytometry data, including frequency features, are expressed in relation to baseline.

The assay used calibrator replicates to estimate the coefficient of variation as a reproducibility metrics. The median coefficient of variation was 5.8% (2.5–10.4%; 10th and 90th percentile) across all plates, indicating excellent assay performance.

### Statistical analysis

For the proteomic analysis, an Elastic Net (EN) regression model was derived for each time point to predict allocation of patients to the respective treatment groups, which selects features while creating mathematical models [28]. One hundred bootstrap iterations were performed during which a subset of the patients equal to the size of the full dataset was randomly selected with replacement. The unpaired two-sided t-test (*p*-value < 0.05) and the AUC were used as criteria to evaluate the performance of the classification models.

The two-sided Mann-Whitney U test (*p*-value < 0.05) was used to identify proteins that differed significantly between the treatment groups. This analysis included all 6,596 unique protein targets. Proteins that differed significantly were included in the pathway analysis and all the proteins were inputted as background. Over-representation pathway enrichment analysis using the Kyoto Encyclopedia of Genes and Genomes (KEGG) database was performed to identify pathways that differed between the two treatment groups [29, 30]. Significance was defined as pathways with an FDR-corrected *p*-value < 0.05. All analyses were performed with GSEApy, Scipy, Numpy, Seaborn and Scikit-learn in Python v2.7.16 [31–33].

The mass cytometry dataset was analyzed using the EN regression method to predict allocation of patients to the respective treatment group [28]. EN models were trained using a Leave-One-Out Cross-Validation (LOOCV) of the AUROC (Area Under the Receiver Operating Characteristic) algorithm [34]. The *p*-values for leave-one-out model predictions were determined with the Mann-Whitney U-test. To evaluate the robustness of feature selected by the EN multivariable models at each time point, a stability selection method was employed. Details regarding the stability selection method and data visualization with the t-distributed Stochastic Neighbor Embedding approach are provided in supplemental materials.

## Results

### Patient characteristics

**Fig. 2:**
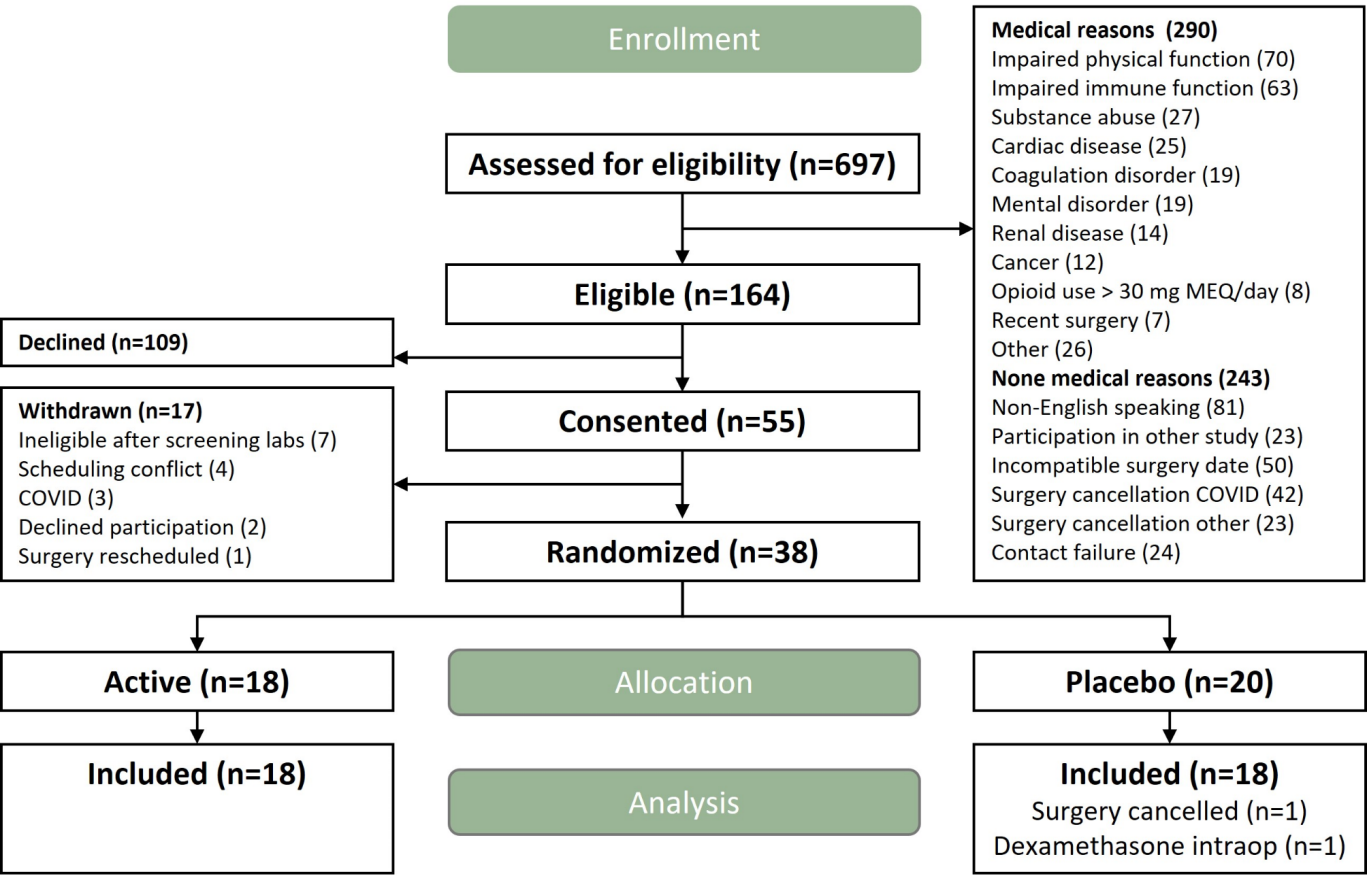
CONSORT chart. Electronic health records of six hundred and ninety-seven patients were screened for study eligibility. Fifty-five of the one hundred and sixty-four eligible patients consented to participate in the study. Seventeen were not suitable for randomization, seven because of laboratory findings meeting exclusion criteria, four because of scheduling challenges, three because of COVID, two because of withdrawn consent, and one because surgery was cancelled. Thirty-eight patients were randomized, but two patients in the placebo group were not included in the primary analysis, one because surgery was cancelled, and the other one because dexamethasone was administered intraoperatively constituting a protocol violation.

Of the 164 eligible patients, 55 consented, and 38 were randomized (Fig. 2). Two patients in the placebo group were not included in the final analysis, one because surgery was cancelled, and one because of the intraoperative administration of steroids, an intervention known to alter the surgical immune response [25]. The demographic and clinical characteristics of enrolled patients were similar between the treatment groups and are summarized in Table 1.

Physical health scores before surgery were below the norm, while mental health scores were within the norm, findings common in the studied surgical population [35, 36]. Evidence for mental depression, anxiety, and stress was absent. The respective median scores were low and in the normal range [37–39].

Surgical and anesthesia-related variables were similar between the groups, including the proportion of patients undergoing hip or knee replacement, and the proportion of patients undergoing general or regional anesthesia (Table 1).

### Modulation of the proteomic response to surgical injury

**Fig. 3:**
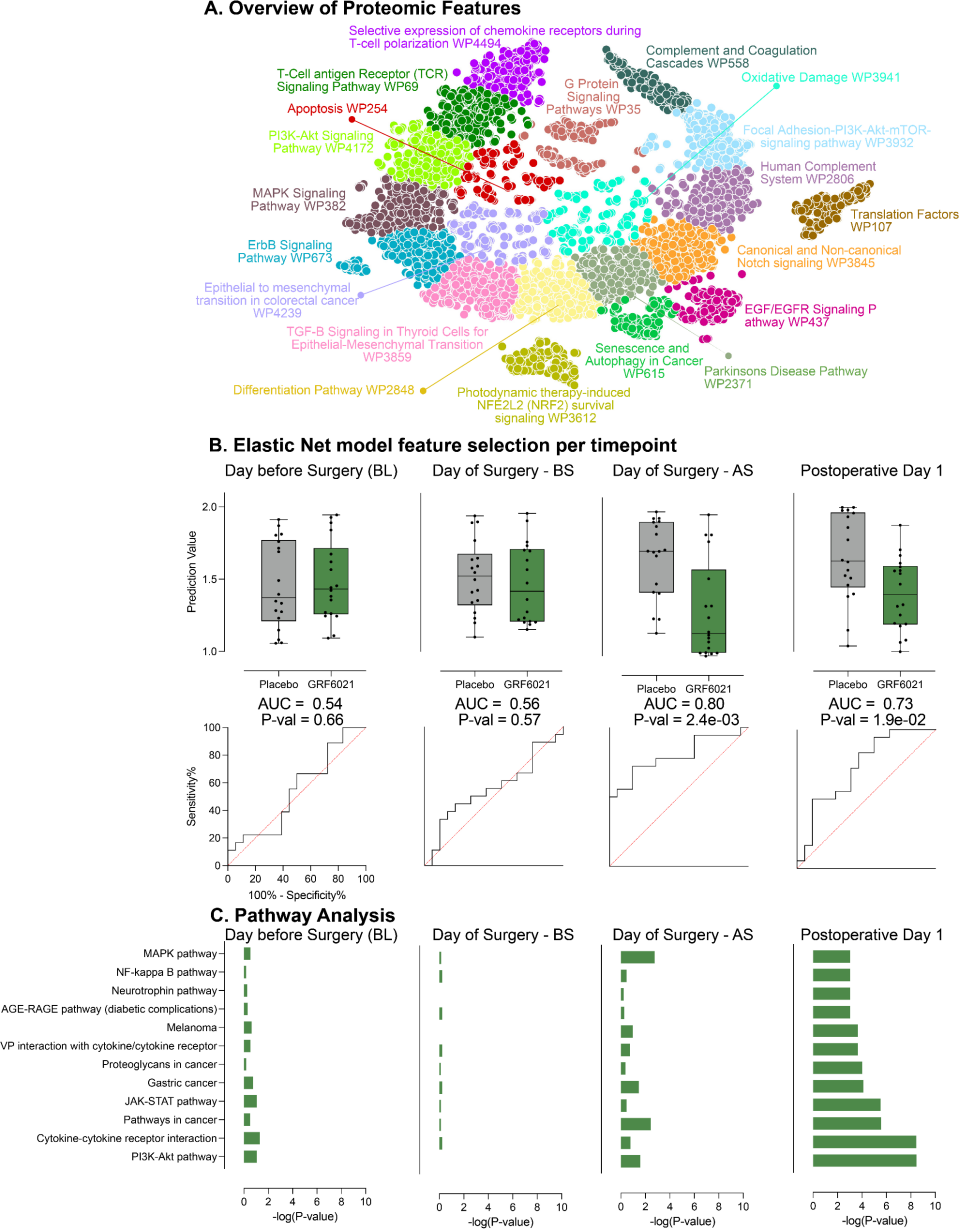
A young plasma protein fraction modulates the proteomic response to surgical injury. (**A**) The dimension of the proteome consisting of 6,596 measured proteins is visualized by a t-SNE plot. Depicted is the proteome as assessed one day before surgery and before starting the infusion of the young plasma protein fraction or placebo. Clusters represent pathways that varied the most among all study participants (k-means algorithm, Wiki Pathways database). (**B**) Box blots depict the Elastic Net classification values for the two treatment groups as the median, interquartile range and whiskers of range. The respective area under the curves (AUC) are shown beneath. While no statistically significant differences were detected one day before surgery and on the day of surgery before surgery (BS), the plasma proteome differed significantly between treatment groups on the day of surgery after surgery (AS) and on postoperative day 1 (two-sided Wilcoxon rank-sum test). (**C**) Pathway analysis using the KEGG database revealed significant enrichment, which was most prominent on postoperative day one. Several of the top-ranking pathways critically regulate inflammatory and immune responses, including the JAK-STAT pathway. The bars depict FDR-corrected -log10 *p*-values.

The dimension of the measured proteome is visualized in Fig. 3A. EN models were trained on the proteomic datasets for each collection time point using a cross-validation and AUROC algorithm. No differences were detected one-day before surgery (AUC = 0.543, *p* = 0.658), or before surgery on the day of surgery (AUC = 0.556, *p* = 0.569). However, the plasma proteomic profile differed significantly between the two treatment groups after surgery on the day of surgery (AUC = 0.796, *p* = 0.002), and on postoperative day 1 (AUC = 0.728, *p* = 0.019) (Fig. 3B). The EN model separating the two treatment groups on the day of surgery after surgery included 195 proteins. The EN model separating the two treatment groups one day after surgery included 554 proteins. The same 43 proteins were included in both models.

Volcano plots depicting the fold-differences between the two treatment groups for all 6,596 protein targets at each time point are shown in Supplementary Fig. 2.

Application of a knowledge-based pathway analysis using the KEGG database highlight signaling pathways that are differentially enriched between the two treatment groups allowed for further biological interpretation of the proteomic EN analysis (Fig. 3C). The Mann-Whitney U test (*p* < 0.05) was used to select proteins for the over-representation analysis. Differences in enrichment were most pronounced on postoperative day 1. Results indicate that administration of the young plasma protein fraction modulated several pathways critically involved in regulating inflammatory and immune responses, including, in rank order, the PI3K-AkT, cytokine-cytokine receptor interaction, JAK-STAT, neurotrophin, NF-kappa B, and MAPK pathways (FDR-corrected *p* < 0.001 for all pathways). For instance, isoforms of class I PI3Ks are expressed in leukocytes and exert pro-inflammatory effects by facilitating the chemokine-mediated recruitment of innate immune cells to the site of inflammation and their activation. In the adaptive branch, class I PI3K isoforms drive normal proliferation and differentiation of naïve CD4^+^ towards the different Th effector subsets [40]. All pathways passing significance at an FDR-corrected *p*-value < 0.05 are listed in Supplementary Table 2.

Plasma proteomics and related pathway analyses are informative at a systems level, but they do not provide insight into cell-type-specific responses. To further investigate the effects of the young plasma protein fraction on targeted pathways, cell-type specific intracellular signaling activities were elucidated with mass cytometry.

### Modulation of the immunological response to surgical injury

**Fig. 4:**
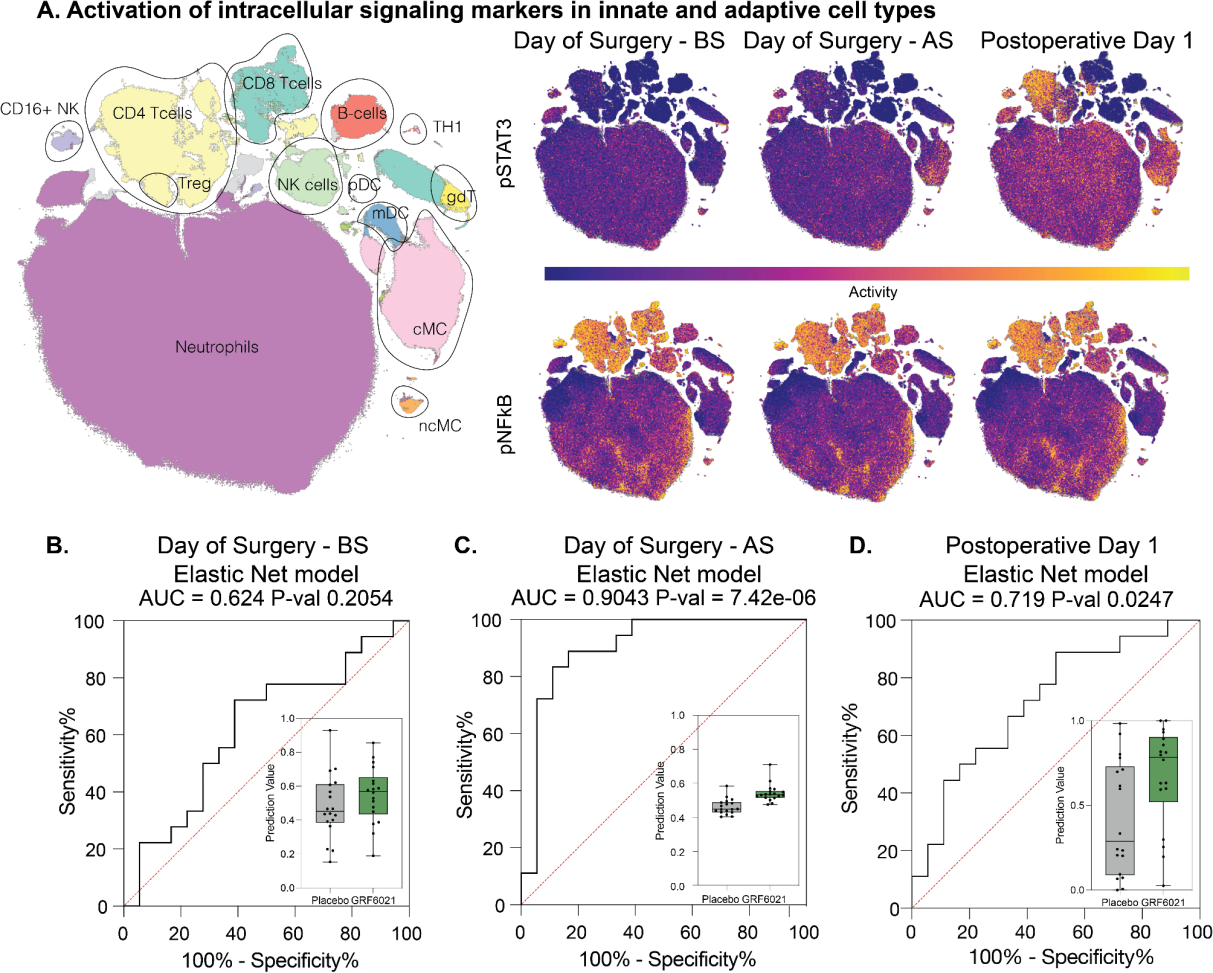
A young plasma protein fraction modulates the immune response to surgical injury. (**A**) A representative tSNE plot depicts manually gated major innate and adaptive immune cell subsets, contoured by black circles. Serial tSNE plots show exemplary cell type-specific signaling activity during the perioperative course. The tSNE colors are scaled to reflect the raw marker expression (low to high). There was a widespread activation of JAK-STAT3 signaling across diverse innate and adaptive immune cell subsets. In contrast, the NFkB signaling remained unchanged. (**B-D**) Box blots depict the Elastic Net classification values for the two treatment groups, namely the median, interquartile range, and whiskers of range at different time points. The respective area under the curve (AUC) is shown in parallel. While no statistically significant differences were detected on the day of surgery before surgery (BS), the immunome was significantly different between the two treatment groups on the day of surgery after surgery (AS), and on postoperative day 1 (two-sided Wilcoxon rank-sum test). NK = natural Killer, Treg = regulatory T cell, pDC = plasmacytoid dendritic cell, mDC = myeloid dendritic cell, gdT = gamma-delta T cell, cMC = classical monocyte, ncMC = non-classical monocyte.

The dimension of the measured immunome and examples of functional changes of the immunome over the course of surgery are visualized in Fig. 4A. Consistent with previous reports, surgical injury caused cell-type specific changes in signaling activity [16, 17, 25]. While EN models did not reveal differences between the two treatment groups before surgery (AUC = 0.624, *p* = 0.205; Fig. 4B), significant differences were detected after surgery on the day of surgery (AUC = 0.904, *p* < 0.001), and one day after surgery (AUC = 0.719, *p* = 0.025; Fig. 4C and D). These results corroborate plasma proteomic findings, indicating that infusion of the young plasma protein fraction modulated the cellular immune response to surgical injury.

**Fig. 5:**
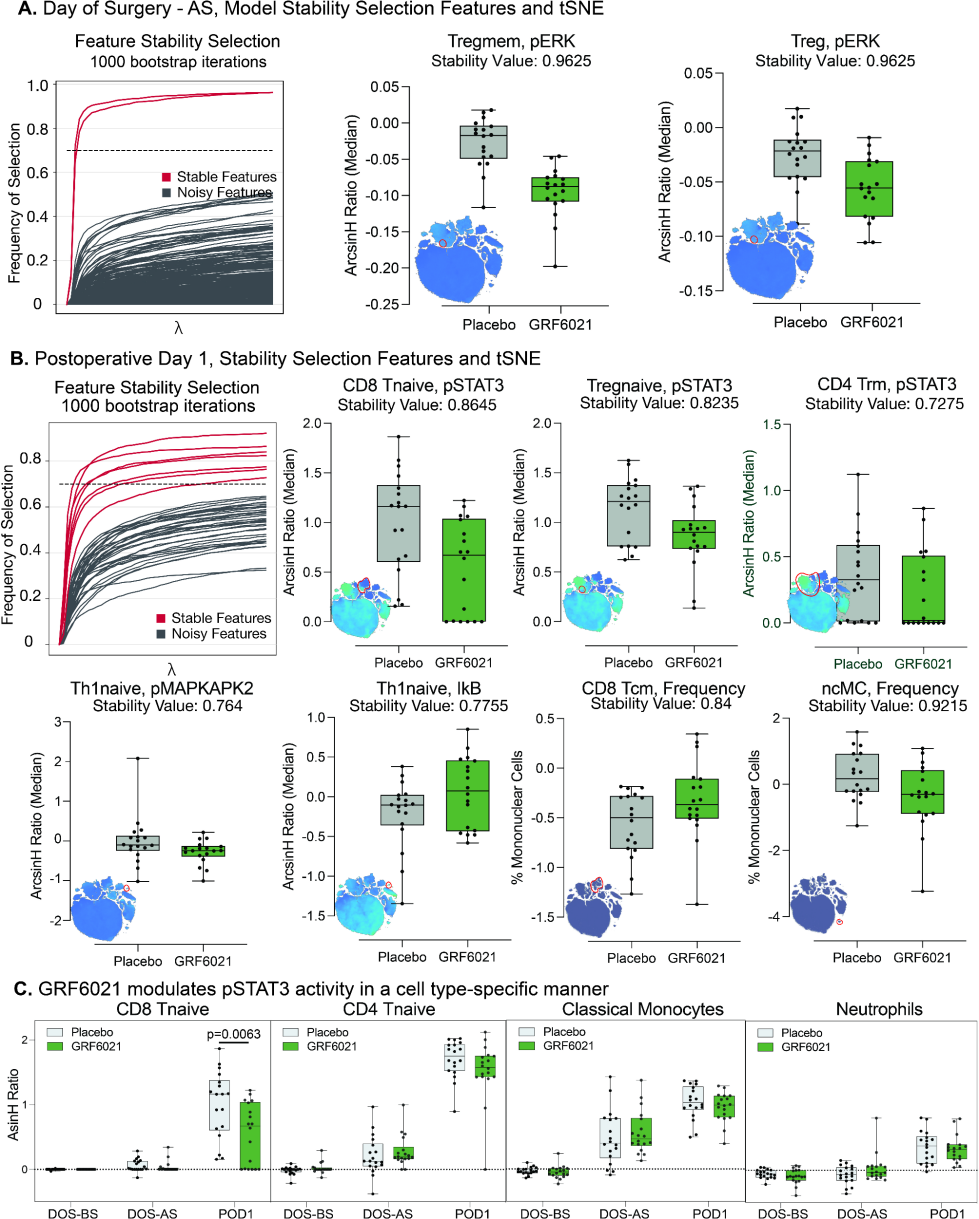
A young plasma protein fraction modulates specific immune cell subsets and signaling pathways after surgical injury. (**A**) Informative (stable) immune features were differentiated from more variable features using a bootstrapping approach with 1,000 iterations and a model inclusion threshold of at least 70%. Two stable features separated the treatment groups one hour after surgery. Phosphorylated extracellular signal-regulated kinase (pERK) activity in regulatory memory T cells (Tregmem) and regulatory T cells (Treg) was lower in patients receiving the young plasma protein fraction when compared to placebo. Box plots depict the median, interquartile range and whiskers of range. The inset tSNE graphs highlight the cell population of interest (red circle). The stability values indicate the frequency of a depicted feature’s selection using the bootstrapping approach (1 = 100%). (**B**) Seven stable features separated the treatment groups on postoperative day 1 (POD1). Phosphorylated signal transducer and activator of transcription 3 (pSTAT3) activity in naïve CD8 naïve T cells (CD8 Tnaive), naïve regulatory T cells (Tregnaive), and resident memory CD4 T cells (CD4 Trm) was lower in patients receiving the young plasma protein fraction. Phosphorylated MAPKAP kinase 2 (pMAPKAPK2) activity was lower in naïve T helper 1 cells (Th1naive), while I-kappa-B (IkB), a regulator of NFkB (nuclear factor kappa-light-chain-enhancer of activated B cells) was higher in these patients. The frequency of central memory CD8 T cells (CD8 Tcm) were increased, while the frequency of non-classical monocytes (ncMC) was decreased in these patients. (**C**) Serial pSTAT3 activity is depicted for naïve CD8 T cells (CD8 Tnaive), naïve CD4 T cells (CD4 Tnaive), classical monocytes, and neutrophils during the perioperative period. The young plasma protein fraction decreased pSTAT3 activity cell-type specifically in naïve CD8 T cells only. *P*-values were derived from two-sided Wilcoxon rank-sum tests.

We used a stability selection algorithm to identify the most robust and informative immune features separating the two treatment groups, to further facilitate the biological interpretation of the EN analyses [41]. Two features separating the treatment groups were particularly eminent after surgery on the day of surgery. pERK signaling in regulator T cells and memory regulatory T cells was lower in patients receiving the young plasma protein fraction (Fig. 5A). Seven features separating the treatment groups were particularly eminent one day after surgery (Fig. 5B). Remarkably, six of these features were signaling responses in adaptive immune cells. Administration of the young plasma protein fraction attenuated pSTAT3 signaling in multiple T cell subsets, including naïve CD8 + T cells, naïve regulatory T cells and resident memory CD4 + T cells. It also attenuated pMAPKAPK2 signaling in naïve T helper cells, while it increased total IkB, a negative regulator of NFkB. Finally, it increased the frequency of central memory CD8 + T cells and decreased the frequency of nonclassical monocytes, a subset of patrolling monocytes responsible for the recruitment of neutrophils and the release of proinflammatory cytokines. Further examination of JAK-STAT3 signaling activity over the course of surgery corroborated that the young plasma protein fraction specifically attenuated STAT3 signaling in adaptive but not in innate immune cells (Fig. 5C).

Taken together, differences in immune cell signaling responses to surgical injury not only confirm the proteomic results highlighting the modulating effects of a young plasma protein fraction on the MAPK and JAK/STAT pathways, but also provide detailed mechanistic information by linking these effects to specific immune cell subsets.

### Clinical outcomes

Directional changes favoring treatment with a young plasma protein fraction were observed for fatigue and associated impairment of daily functioning, as well as pain (Supplementary Fig. 3). While observed differences were not statistically significant, the median time to 50% recovery from fatigue and impairment of daily functioning was 12 days (IQR, 6–16) in the active treatment group, and 16 days (IQR, 7–22) in the placebo group. The median time to mild pain was 12 days (IQR, 11–18) in the active treatment group, and 18 days (IQR, 9–30) in the placebo group. While the median cumulative opioid consumption during the 6-week observation period after hospital discharge was similar, opioid consumption was lower in the active treatment group during hospitalization (Table 1). The median consumption on postoperative days 1 and 2 was 2.3 mg (IQR, 1.9–3.1) and 1.9 mg (IQR, 0.9–4.1), compared to 3.4 mg (IQR, 1.7–5.2) and 2.3 mg (1.8–4.5). Visual inspection of Supplementary Fig. 3 suggests that group differences were most pronounced when considering the upper range of the interquartile range. While speculative, this observation is compatible with the view that patients at risk for a prolonged and impaired recovery may benefit most from the active intervention.

To address the question whether clinical outcomes were associated with significant immune features separating the two treatment groups a correlation analysis was performed. This analysis did not not reveal any significant results passing multiple comparison correction.

## Discussion

This study sought to determine whether administering a young plasma protein fraction in an elderly population affects archetypical inflammatory and immune responses using surgery as a controlled human injury model. The administration of the young plasma protein fraction resulted in significant signaling pathway- and cell type-specific anti-inflammatory immune modulation. To the best of our knowledge, this is the first study in humans providing evidence that components in young plasma possess immune-modulating properties in an elderly population. These findings are relevant given the ongoing debate regarding the potential health benefits of young plasma for elderly humans, which is currently lacking data [1–3].

Sentinel inflammatory pathways modulated by the administration of the young plasma protein fraction included the PI3K-AkT, cytokine-cytokine receptor interaction, JAK-STAT, neurotrophin, NF-kappa B, and MAPK pathways. These results echo preclinical findings indicating that some beneficial effects observed in parabiosis and plasma transfer models are linked to the regulation of inflammatory pathways [7–10]. Notably, a significant number of the enriched pathways serve broader biological functions beyond the regulation of inflammatory responses. For example, the top-ranked pathway PI3K-AkT is mediating numerous cellular functions including angiogenesis, metabolism, growth, proliferation, and aptosis [42, 43]. Similarly, several enriched pathways were linked to cancer biology. The finding that a young plasma protein fraction regulated cancer-related pathways is plausible, given that a major risk factor for developing cancer is an age-related progressive decline in physiological integrity [15, 44]. In this context, a recent multiomic study in mice using the heterochronic parabiosis model and identifying several of the enriched pathways discussed here is noteworthy. Exposure to the young circulating system induced persistent and systemic slowing of epigenetic aging, culminating in an extension of lifespan [45]. Taken together, the proteomic findings suggest that the administration of a young plasma protein fraction in elderly humans may influence a broad array of biological processes potentially associated with aging and age-related disease burden.

Complementing and expanding on the proteomic dataset, mass cytometry provided a single-cell read-out of the modulating effects of the young plasma protein fraction on immune cells in response to surgical injury. The young plasma protein fraction preferentially attenuated cell type-specific signaling responses in the adaptive immune branch. Specifically, the MAPK and JAK/STAT3 signaling responses in CD4 + and CD8 + T cells and regulatory T cell subsets were dampened, echoing the results of the proteomic pathway enrichment analysis. Notably, while the young plasma protein fraction exerted profound inhibitory effects on STAT3 signaling responses in T cell subsets, such effects were not observed in innate immune cells.

The JAK/STAT3 signaling pathway orchestrates a multi-faceted program in acute inflammation by striking a balance between effective pathogen defense, initially driven by innate immune cells, and immuno-regulatory mechanisms required for tissue repair, wound healing, and functional recovery [17, 26, 46–48]. Notably, a recent study of elderly patients reported enhanced JAK/STAT3 signaling responses in CD4 + and CD8 + T cell in patients at greater risk for cognitive decline after surgery [49]. A such, factors present in young plasma may attenuate inflammaging-associated inflammatory responses mediated by the adaptive immune branch, while preserving innate mechanisms required for pathogen defense and tissue repair.

In the context of chronic inflammation, sustained activation of JAK/STAT3 signaling by canonical pro-inflammatory cytokines, including IL-6, is a biological hallmark of inflammaging and is linked to several age-associated illnesses, including cardiovascular diseases, neurocognitive disorders, and cancer [50–53]. The dampening of JAK/STAT3 responses in T cell subsets corroborate the proteomic results indicating that the young plasma protein fraction regulated pathways related to cancer.

Age is associated with profound changes in the plasma proteome, which are adeptly described by a proteomic aging clock [54, 55]. An acceleration of the aging clock is associated with an increase in disease burden and mortality [56]. These findings support the search for rejuvenating plasma factors capable of reversing age-related biological processes, as well as pro-aging factors accelerating these processes [57]. Several rejuvenating factors have been identified, including apelin, tissue inhibitor of metalloproteinases 2, and platelet-derived factor 4, which exert beneficial effects on mitochondriogenesis, neurogenesis, regulation of neuroinflammation, and cognitive function in aged mice [11, 58–61]. Growth differentiation factor 11 (GRF11) is another rejuvenating factor, and its recombinant form is in clinical development for improving stroke-recovery and acute inflammatory conditions, including pancreatitis. Preclinical results considering its beneficial effects are mixed, though [62–67].

A group of investigators suggested that removing pro-aging factors in blood suffices to rejuvenate old tissue [68–70]. While there are known plasma factors that promote biological aging including β2-microglobulin and CCL11, a significant body of evidence highlights the biological relevance of rejuvenating factors in experimental protocols unlikely to dilute pro-aging factors in blood plasma [59–61, 63, 65, 71, 72]. Based on our current understanding, therapeutic approaches that aim to augment rejuvenating factors or inhibit pro-aging factors and their receptors are both worthwhile strategies.

The average age of plasma donors was 35 years. The question arises whether a larger effect size could be observed when plasma from younger subjects, including umbilical cord plasma, is used. A study in mice focusing on cognitive function reported larger effect sizes after the administration of human umbilical cord plasma compared to plasma from young adults [11], making it plausible that effect sizes increase when administering protein fractions that mirror the active principles present in umbilical cord plasma.

Other control interventions than normal saline could have been considered within the context of this study. The selection of the control intervention is largely contingent upon the primary inquiry the study aims to address, and each selection carries inherent limitations regarding the conclusions that can be drawn. We chose normal saline as the control intervention to address the basic question whether administration of a human protein fraction modulates the inflammatory and immune response to surgery when compared to a solution that does not contain any proteins and only few solvents to render it isotonic. There are inherent limitations to using normal saline as a control intervention, including the uncertainty as to which proteins mediated the observed effects and whether similar effects could be observed when using plasma fractions from older donors.

## Conclusion

The current results are a first proof of principle in humans, indicating that factors present in young plasma regulate biological processes that are linked to age-related pathologies. They provide a solid rationale for further elucidating the active principles in young plasma that could benefit the treatment of age-related pathologies.

## Electronic supplementary material

Below is the link to the electronic supplementary material.

Supplementary Material 1

## Data Availability

The data sets generated and analyzed during the current study are available on Figshare: 10.6084/m9.figshare.25513426. The codes used for analyses are available on GitHub: https://github.com/MaxSabayev/AlkahestRepo.
