# Supplementary material for: Infusion of young donor plasma components in older patients modifies the immune and inflammatory response to surgical tissue injury: a randomized clinical trial
Source: J Transl Med. 2025 Feb 14;23:183. doi: 10.1186/s12967-025-06215-w (PMC11829456; doi:10.1186/s12967-025-06215-w)
Supplement: Supplementary file 1 — Supplementary Material 1 [file 12967_2025_6215_MOESM1_ESM.docx]

**Supplementary Material**

**Methods**

**Patient inclusion and exclusion criteria**

Inclusion criteria were: (1) scheduled to undergo primary total hip or knee arthroplasty, (2) age between 50-85 years, (3) glomerular filtration rate ≥ 45 mL/min, (4) women not pregnant or breastfeeding, (5) women of childbearing potential with negative pregnancy test and agreeable to use acceptable forms of contraception before study entry, (6) understand and speak English and able to follow study instructions, (7) signed informed consent form.

Exclusion criteria were: (1) blood coagulation disorders, (2) started on chronic anticoagulant therapy during the last 6 months if not related to surgery, (3) diseases associated with a hypercoagulable state, (4) deep vein thrombosis or pulmonary embolism during the last 6 months, (5) disseminated intravascular coagulation, thrombotic thrombocytopenic purpura, or heparin-induced thrombocytopenia during the last 5 years, (6) hypersensitivity reaction to any human blood product, (7) treatment with human blood product during the last 6 months, (8) immunoglobulin A or haptoglobin deficiency, (9) complications to intravenous immunoglobulin therapy, (10) major surgery, trauma or injury during the last 3 months or minor surgery during the last month, (11) unstable coronary heart disease during the last 6 months, (12) congestive heart failure NYHA class III or IV, or associated with a metabolic equivalent < 4, (13) arterial hypertension with systolic blood pressure ≥ 170 mmHg and/or diastolic blood pressure ≥ 110 mmHg, (14) current treatment with ≥ 3 antihypertensive medications, (15) clinically significant abnormalities on electrocardiogram, (16) three-fold elevation of liver enzymes (AST and ALT), (17) clinically significant abnormalities in complete blood count, complete metabolic, and/or coagulation panel, (18) major infection (e.g., pneumonia) during the last 3 months, or minor infection (e.g., UTI) during the last month, (19) hemoglobin < 8 g/dL, (20) urine protein-to-creatinine ratio > 1.5, (21) inadequate venous access for reliable drug delivery or blood draws, (22) concurrent participation in other interventional trial, (23) diseases associated with significant alterations in immune function, (24) concurrent immunosuppressant therapy, (25) substance abuse during the last 2 years, (26) functional impairment of lower extremity unrelated to surgical target joint, (27) any other condition that in the opinion of the investigator may interfere with the safety of a study participant, the study conduct, or the interpretation of study data.

**Clinical outcomes**

The main clinical outcomes fatigue and associated impairment of daily functioning, pain and associated opioid consumption, and functional impairment of the affected leg were assessed one day before surgery, and on postoperative day one and two while hospitalized. After discharge, these outcomes were assessed remotely under the guidance of a study coordinator on postoperative days 4, 7, 10, 14, 17, 21, 28, 35, and 42. Screening for delirium was performed one day before surgery, and on postoperative days 1 and 2.

Fatigue and associated impairment of daily functioning was assessed with the Surgical Recovery Scale (SRS) (1). The SRS is sensitive to change and correlates with the course and severity of postoperative complications (2, 3). SRS scores range between 17-100 (worst - best). Speed of recovery was quantified as the time required to half-maximum recovery equivalent to: lowest postoperative SRS score + ((baseline SRS score - lowest postoperative SRS score)/2).

Pain and function of the operated leg were assessed with an adapted and validated version of the WOMAC scale (4). Patients rated pain at night, at rest, while weight-bearing, and while walking on a flat surface on 11-point numerical scales, resulting in a compound score between 0-40 (no to worst pain). Similarly, patients rated functional impairment of the operated leg while lying, sitting, raising from the bed, raising from the chair, standing, and walking on a flat surface, resulting in a compound score from 0 to 60 (no to worst impairment). Recovery from pain was defined as the time required to consistently report a score of less than 12 reflecting the transition from moderate to mild pain. Recovery from functional impairment of the operated leg was defined as the time required to report a score consistently less than 18 reflecting the transition from moderate to mild impairment.

Opioid consumption was quantified as the daily intake of intravenous hydromorphone equivalents (mg), using a conversion table to allow for group comparison (5). Presence or absence of delirium was determined with the 3-Minute Diagnostic Interview for Confusion Assessment Method (6). Presence and severity of depression was assessed with the Beck Depression Inventory-II (BDI-II), scores of 0-13, 14-19, 20-28, and >28 indicating no, mild, moderate and severe depression, respectively (7). Mental and physical health was assessed with the Short Form Health Survey 36 (8). Results are expressed as z-scores with a population mean of 50 and a standard deviation of 10. Anxiety was assessed with the Profile of Mood States with scores ranging between 0-36 (9). Normative data are available for score interpretation (10). Frequency and severity of stress during the last month was quantified with the 10-item stress scale (11). The compound score ranges from 0-40.

**Study size considerations**

The primary outcome of this study was a difference in the inflammatory and immunological signatures between the two treatment groups using a multivariate modelling approach, which prevents a traditional power analysis and sample size determination. However, a sample size of about 2 x 20 subjects was sufficient to detect relevant differences in a previous and analogous study examining interventions expected to have a similar effect size (12). Due to the significant logistical and recruitment obstacles posed by COVID, a total of 38 patients instead of the intended 40 were enrolled in this study.

**Proteomic assay**

**S**amples were randomly allocated and incubated on 96-well plates with a mixture of SOMAmer reagents. Two sequential bead-based immobilization and washing steps were used to eliminate non-specifically bound proteins, unbound proteins, and unbound SOMAmer reagents from protein target-bound reagents. After eluting SOMAmer reagents from the target proteins, the fluorescence-labeled reagents were quantified on an Agilent hybridization array (Agilent Technologies, Santa Clara, CA). Data were normalized in four specific steps and according to assay data quality control procedures defined in the good laboratory practice quality system of SomaLogic. Normalization steps control for signal intensity biases introduced by differential hybridization efficiencies and the overall brightness of plates, collection protocol artifacts, and batch effects between different plates. Quality control was passed by 98.7% of samples.

The proteomics data was visualized using a correlation matrix encompassing all measured proteins. The tSNE (t-distributed Stochastic Neighbor Embedding) algorithm used this matrix to calculate a local embedding, which facilitates the comprehension of the data structure in a reduced dimensionality. To identify homogeneous groups of proteins that exhibit similar abundance patterns across diverse patients, clustering analysis was conducted employing the k-means algorithm with default parameters. Significantly enriched pathways were identified by FDR-corrected p-values < 0.05. The most enriched pathways representing respective clusters were used to annotate clusters of proteins. The KEGG database provided the foundation for pathway enrichment analysis.

**Mass cytometry**

For barcoding, six palladium isotopes were used in combinations of three for barcoding, allowing analyzing twenty samples per plate. Serial samples from both treatment groups were gender and age-matched and analyzed simultaneously using the Helios system to maximize the detection of group-differences. The FCS output files were normalized (SOURCE) and debarcoded (13, 14).

The stability selection algorithm builds, for several hyperparameter sets, models on a number B = 2000 of bootstraps of the dataset (15). This allows calculating, for a given hyperparameter set, the selection frequency of each model feature. In other words, this approach reveals the percentage of bootstraps in which a particular feature was included in the model, i.e., was associated with a non-null coefficient. Taking the selection frequency for each feature over all hyperparameter sets reveals the stability score, a score equal of 1 indicating that a feature was selected in all bootstraps. The stability selection models yielded sets of features ranked by relative importance (stability score). EN features with the highest frequency of selection (>70%) were examined further.

To complement the manually gated data, unsupervised non-linear dimensionality reduction technique, t-SNE (t-distributed Stochastic Neighbor Embedding), was employed using 26 phenotypic markers. Expression of arcsinh transformed intracellular signaling markers were projected across the timepoints and scaled per single marker to enhance marker-specific responses to surgical trauma. Additional box-and-whisker and AUROC graphs were created in Prism.

**References**

1. Paddison JS, Sammour T, Kahokehr A, Zargar-Shoshtari K, Hill AG. Development and validation of the Surgical Recovery Scale (SRS). The Journal of surgical research. 2011;167(2):e85-91.

2. Singh PP, Srinivasa S, Lemanu DP, Kahokehr AA, Hill AG. The Surgical Recovery Score correlates with the development of complications following elective colectomy. The Journal of surgical research. 2013;184(1):138-44.

3. Ganio EA, Stanley N, Lindberg-Larsen V, Einhaus J, Tsai AS, Verdonk F, et al. Preferential inhibition of adaptive immune system dynamics by glucocorticoids in patients after acute surgical trauma. Nature communications. 2020;11(1):3737.

4. Gaudilliere B, Fragiadakis GK, Bruggner RV, Nicolau M, Finck R, Tingle M, et al. Clinical recovery from surgery correlates with single-cell immune signatures. Science translational medicine. 2014;6(255):255ra131.

5. McAuley DF. Opioid Analgesic Converter <http://www.globalrph.com/narcoticonv.htm>: Global RPh; 2017 [updated 09/07/2017. Available from: <http://www.globalrph.com/narcoticonv.htm>.

6. Marcantonio ER, Ngo LH, O'Connor M, Jones RN, Crane PK, Metzger ED, Inouye SK. 3D-CAM: derivation and validation of a 3-minute diagnostic interview for CAM-defined delirium: a cross-sectional diagnostic test study. Ann Intern Med. 2014;161(8):554-61.

7. Beck A, Steer R, Brown G. Beck Depression Inventory - Manual. 2nd ed. San Antonio: The Psychological Corporation; 1996.

8. Patel AA, Donegan D, Albert T. The 36-item short form. The Journal of the American Academy of Orthopaedic Surgeons. 2007;15(2):126-34.

9. McNair DM, Loor M, Droppelmann LF. Profile of mood states. San Diego, CA: Educational and Industrial Testing Service; 1992.

10. Nyenhuis DL, Yamamoto C, Luchetta T, Terrien A, Parmentier A. Adult and geriatric normative data and validation of the profile of mood states. J Clin Psychol. 1999;55(1):79-86.

11. Cohen S, Kamarck T, Mermelstein R. A global measure of perceived stress. Journal of health and social behavior. 1983;24(4):385-96.

12. Aghaeepour N, Kin C, Ganio EA, Jensen KP, Gaudilliere DK, Tingle M, et al. Deep Immune Profiling of an Arginine-Enriched Nutritional Intervention in Patients Undergoing Surgery. J Immunol. 2017.

13. Finck R, Simonds EF, Jager A, Krishnaswamy S, Sachs K, Fantl W, et al. Normalization of mass cytometry data with bead standards. Cytometry Part A : the journal of the International Society for Analytical Cytology. 2013;83(5):483-94.

14. Zunder ER, Finck R, Behbehani GK, Amir el AD, Krishnaswamy S, Gonzalez VD, et al. Palladium-based mass tag cell barcoding with a doublet-filtering scheme and single-cell deconvolution algorithm. Nature protocols. 2015;10(2):316-33.

15. Meinshausen N, Bühlmann P. Stability selection. Journal of the Royal Statistical Society Series B: Statistical Methodology. 2010;72(4):417-73.

**Supplementary Table 1.**  Antibody panel

**Supplementary Table 2.**  Pathways with FDR-corrected p-value < 0.05

| **Time point: Day of surgery after surgery** |  |  |
| --- | --- | --- |
|  |  |  |
| **Pathway** | **Raw p-value** | **FDR-corrected p-value** |
| MAPK signaling pathway | 7.60E-06 | 1.74E-03 |
| Pathways in cancer | 4.07E-05 | 3.60E-03 |
| Axon guidance | 4.71E-05 | 3.60E-03 |
| Ras signaling pathway | 3.45E-04 | 1.69E-02 |
| Transcriptional misregulation in cancer | 3.70E-04 | 1.69E-02 |
| Rap1 signaling pathway | 7.07E-04 | 2.60E-02 |
| PI3K-Akt signaling pathway | 7.93E-04 | 2.60E-02 |
| Glycosaminoglycan degradation | 1.24E-03 | 3.45E-02 |
| PPAR signaling pathway | 1.57E-03 | 3.45E-02 |
| Gastric cancer | 1.63E-03 | 3.45E-02 |
| Chemokine signaling pathway | 1.66E-03 | 3.45E-02 |
| Ovarian steroidogenesis | 2.70E-03 | 4.77E-02 |
| Hippo signaling pathway | 2.71E-03 | 4.77E-02 |
|  |  |  |
| **Time point: One day after surgery** |  |  |
|  |  |  |
| **Pathway** | **Raw p-value** | **FDR-corrected p-value** |
| PI3K-Akt signaling pathway | 1.19E-11 | 3.41E-09 |
| Cytokine-cytokine receptor interaction | 2.58E-11 | 3.68E-09 |
| Pathways in cancer | 2.92E-08 | 2.78E-06 |
| JAK-STAT signaling pathway | 4.26E-08 | 3.04E-06 |
| Gastric cancer | 1.35E-06 | 7.71E-05 |
| Proteoglycans in cancer | 1.96E-06 | 9.35E-05 |
| Viral protein interaction with cytokine and cytokine receptor | 5.56E-06 | 2.15E-04 |
| Melanoma | 6.02E-06 | 2.15E-04 |
| AGE-RAGE signaling pathway in diabetic complications | 2.89E-05 | 9.17E-04 |
| Neurotrophin signaling pathway | 3.72E-05 | 9.52E-04 |
| NF-kappa B signaling pathway | 4.28E-05 | 9.52E-04 |
| MAPK signaling pathway | 4.33E-05 | 9.52E-04 |
| Kaposi sarcoma-associated herpesvirus infection | 4.29E-05 | 9.52E-04 |
| IL-17 signaling pathway | 7.79E-05 | 1.59E-03 |
| Breast cancer | 8.67E-05 | 1.63E-03 |
| Human papillomavirus infection | 9.10E-05 | 1.63E-03 |
| FoxO signaling pathway | 1.01E-04 | 1.65E-03 |
| Prostate cancer | 1.04E-04 | 1.65E-03 |
| Bladder cancer | 1.47E-04 | 2.13E-03 |
| ErbB signaling pathway | 1.57E-04 | 2.13E-03 |
| Lipid and atherosclerosis | 1.63E-04 | 2.13E-03 |
| Amoebiasis | 1.64E-04 | 2.13E-03 |
| MicroRNAs in cancer | 2.57E-04 | 3.19E-03 |
| Shigellosis | 2.77E-04 | 3.30E-03 |
| HIF-1 signaling pathway | 2.95E-04 | 3.37E-03 |
| Chronic myeloid leukemia | 3.15E-04 | 3.47E-03 |
| Natural killer cell mediated cytotoxicity | 3.91E-04 | 4.14E-03 |
| Thyroid cancer | 5.88E-04 | 6.00E-03 |
| Glycolysis / Gluconeogenesis | 6.38E-04 | 6.29E-03 |
| Fc epsilon RI signaling pathway | 7.05E-04 | 6.72E-03 |
| Renal cell carcinoma | 7.78E-04 | 6.80E-03 |
| Colorectal cancer | 7.91E-04 | 6.80E-03 |
| T cell receptor signaling pathway | 8.05E-04 | 6.80E-03 |
| Apoptosis | 8.08E-04 | 6.80E-03 |
| ECM-receptor interaction | 9.35E-04 | 7.64E-03 |
| Non-small cell lung cancer | 1.03E-03 | 8.21E-03 |
| Ras signaling pathway | 1.12E-03 | 8.62E-03 |
| Small cell lung cancer | 1.28E-03 | 9.67E-03 |
| Glioma | 1.35E-03 | 9.91E-03 |
| GnRH signaling pathway | 1.39E-03 | 9.92E-03 |
| VEGF signaling pathway | 1.43E-03 | 1.00E-02 |
| Pancreatic cancer | 1.47E-03 | 1.00E-02 |
| Cellular senescence | 1.82E-03 | 1.21E-02 |
| Choline metabolism in cancer | 2.00E-03 | 1.29E-02 |
| Focal adhesion | 2.03E-03 | 1.29E-02 |
| Human cytomegalovirus infection | 2.19E-03 | 1.30E-02 |
| B cell receptor signaling pathway | 2.22E-03 | 1.30E-02 |
| Growth hormone synthesis, secretion and action | 2.24E-03 | 1.30E-02 |
| Sphingolipid signaling pathway | 2.24E-03 | 1.30E-02 |
| Fructose and mannose metabolism | 2.32E-03 | 1.30E-02 |
| Axon guidance | 2.32E-03 | 1.30E-02 |
| Hepatitis B | 2.50E-03 | 1.37E-02 |
| Thyroid hormone signaling pathway | 2.54E-03 | 1.37E-02 |
| Malaria | 2.94E-03 | 1.56E-02 |
| Rap1 signaling pathway | 3.03E-03 | 1.58E-02 |
| Parathyroid hormone synthesis, secretion and action | 3.42E-03 | 1.75E-02 |
| Chemokine signaling pathway | 3.68E-03 | 1.82E-02 |
| Transcriptional misregulation in cancer | 3.68E-03 | 1.82E-02 |
| Central carbon metabolism in cancer | 3.85E-03 | 1.85E-02 |
| Protein processing in endoplasmic reticulum | 3.88E-03 | 1.85E-02 |
| PD-L1 expression and PD-1 checkpoint pathway in cancer | 4.00E-03 | 1.86E-02 |
| Purine metabolism | 4.03E-03 | 1.86E-02 |
| p53 signaling pathway | 4.86E-03 | 2.20E-02 |
| TNF signaling pathway | 4.92E-03 | 2.20E-02 |
| Legionellosis | 5.67E-03 | 2.49E-02 |
| Endometrial cancer | 6.17E-03 | 2.68E-02 |
| Endocytosis | 6.28E-03 | 2.68E-02 |
| Inflammatory mediator regulation of TRP channels | 7.13E-03 | 3.00E-02 |
| Chagas disease | 9.01E-03 | 3.73E-02 |
| Hepatocellular carcinoma | 9.22E-03 | 3.77E-02 |
| Inflammatory bowel disease | 1.07E-02 | 4.29E-02 |
| Cardiac muscle contraction | 1.24E-02 | 4.93E-02 |

**Supplementary Figure 1.** Gating strategy

**
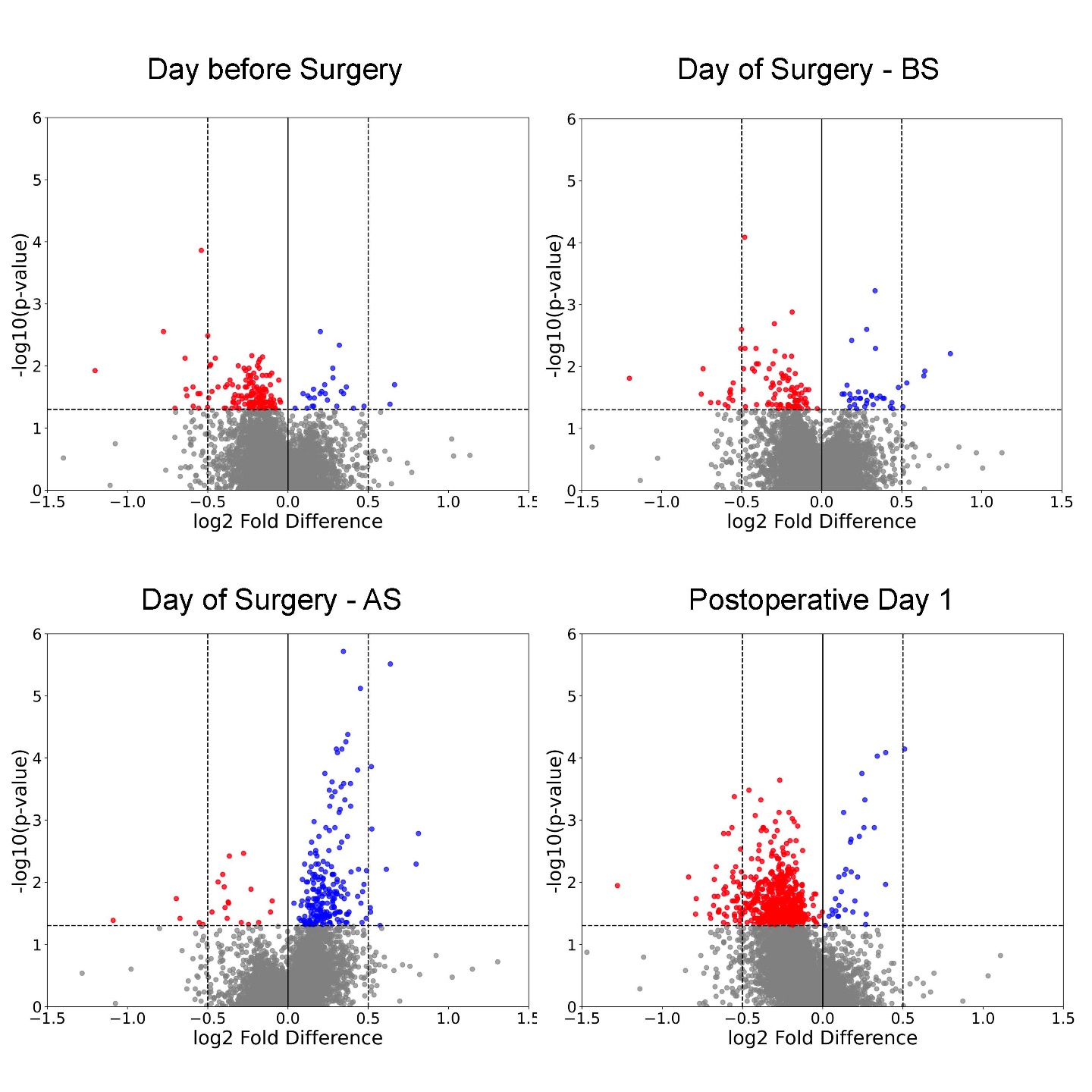
Supplementary Figure 2.**

**Volcano plots** – Depicted are the fold-differences (x-axis) between the two treatment groups for all 6,596 protein targets at each time point. Positive differences indicate that the protein level was higher in the treatment group that received the young plasma protein fraction, whereas negative differences indicate that the levels were lower in this group. These differences are plotted against the p-value (y-axis) associated with each protein (p<0.05, two-sided Mann-Whitney U test). Red dots depict proteins that were higher in the group receiving the young plasma protein fraction (p<0.05, Mann-Whitney U test). Blue dots depict proteins that were higher in the group receiving saline placebo. Visual examination of the plots indicates that the differences between the two treatment groups were more pronounced after surgery compared to one day before surgery and the day of surgery before surgery (BS). Notably, most proteins that separated the two groups on the day of surgery after surgery (AS) were elevated in patients receiving saline placebo. Conversely, most proteins that separated the two groups one day after surgery (AS) were elevated in patients receiving the young plasma protein fraction.

**Supplementary Figure 3**

**
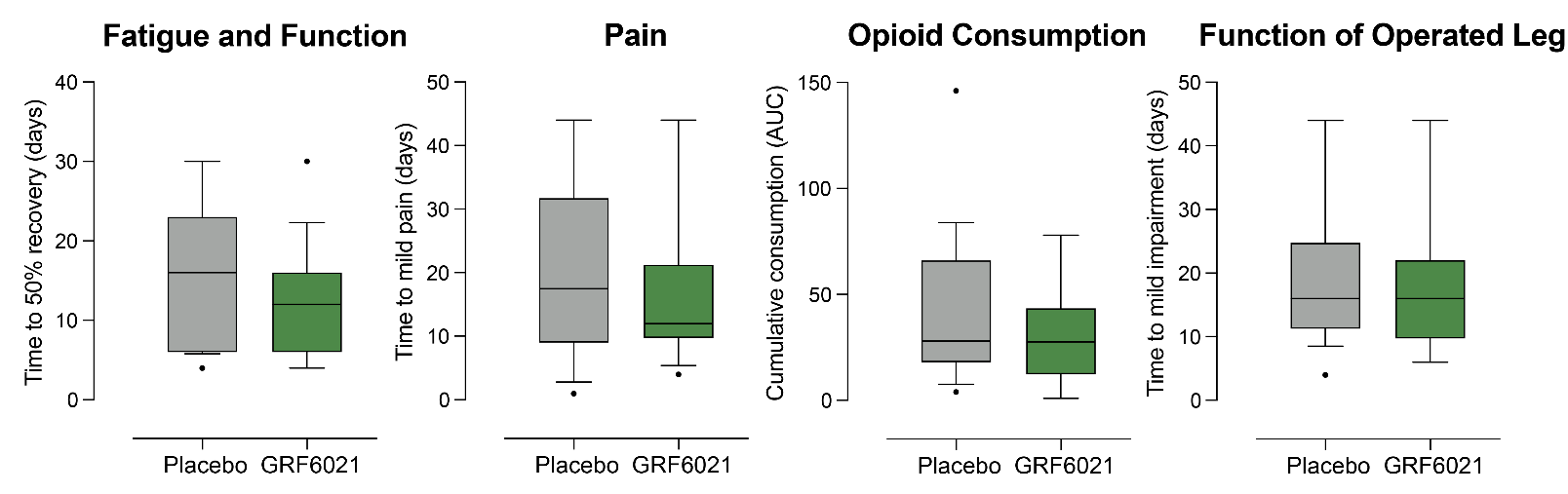
**

**Major clinical outcomes** - Directional changes towards more favorable clinical outcomes were observed in patients randomized to the young plasma protein fraction (GRF6021). Clinical outcomes were not significantly different between treatment groups. Box plots depicting the interquartile ranges and 90% confidence intervals suggest that fatigue and associated impairment of daily functioning, and pain and associated cumulative opioid consumption were lower in patients receiving GRF6021. The cumulative opioid consumption is the total quantity of IV hydromorphone equivalents (mg) consumed during the six-week observation period following hospital discharge. No differences were apparent for the functioning of the operated leg.
